# Complications of Decompressive Craniectomy

**DOI:** 10.3389/fneur.2018.00977

**Published:** 2018-11-20

**Authors:** M. S. Gopalakrishnan, Nagesh C. Shanbhag, Dhaval P. Shukla, Subhas K. Konar, Dhananjaya I. Bhat, B. Indira Devi

**Affiliations:** ^1^Department of Neurosurgery, Jawaharlal Institute of Postgraduate Medical Education and Research, Puducherry, India; ^2^Department of Neurosurgery, National Institute of Mental Health and Neurosciences, Bangalore, India; ^3^NIHR Global Health Research Group on Neurotrauma, University of Cambridge, Cambridge, United Kingdom

**Keywords:** decompressive craniectomy, hemorrhage expansion, infections, cerebral herniation, seizures, hydrocephalus, syndrome of the trephined

## Abstract

Decompressive craniectomy (DC) has become the definitive surgical procedure to manage medically intractable rise in intracranial pressure due to stroke and traumatic brain injury. With incoming evidence from recent multi-centric randomized controlled trials to support its use, we could expect a significant rise in the number of patients who undergo this procedure. Although one would argue that the procedure reduces mortality only at the expense of increasing the proportion of the severely disabled, what is not contested is that patients face the risk of a large number of complications after the operation and that can further compromise the quality of life. Decompressive craniectomy (DC), which is designed to overcome the space constraints of the Monro Kellie doctrine, perturbs the cerebral blood, and CSF flow dynamics. Resultant complications occur days to months after the surgical procedure in a time pattern that can be anticipated with advantage in managing them. New or expanding hematomas that occur within the first few days can be life-threatening and we recommend CT scans at 24 and 48 h postoperatively to detect them. Surgeons should also be mindful of the myriad manifestations of peculiar complications like the syndrome of the trephined and neurological deterioration due to paradoxical herniation which may occur many months after the decompression. A sufficiently large frontotemporoparietal craniectomy, 15 cm in diameter, increases the effectiveness of the procedure and reduces chances of external cerebral herniation. An early cranioplasty, as soon as the brain is lax, appears to be a reasonable choice to mitigate many of the late complications. Complications, their causes, consequences, and measures to manage them are described in this chapter.

## Introduction

In medicine, there is increasing awareness that outcome must be evaluated in terms of quality of life and cost effectiveness, rather than merely extending the survival of a patient. Such considerations are especially important in decompressive craniectomy (DC), which is performed in certain cases of ischemic stroke, traumatic brain injury, and subarachnoid hemorrhage, to alleviate (ICP) and massive brain swelling (1–3). ICP reduction can lead to improvements in cerebrovascular compliance, cerebral oxygenation, and cerebral perfusion (4). Though many studies have shown long-term beneficial effects after DC (1, 5–7) it is still regarded as a salvage surgery. Long-term, deleterious neurocognitive, and psychosocial effects resulting in poor quality of life, and economical burden are well known (6, 8).

Anticipating a possible rise in the frequency with which decompressive craniectomies are likely to be carried out, based on the strength of recent, strong, supportive, level-one evidence in both traumatic brain injury (9) and stroke (10, 11), complication avoidance should become the new focus in surgical management and research. Currently, there is only low-quality evidence to choose the kind of interventions to avoid complications. Understanding the type and burden of the potential complications, the timeline of their appearance and the reasons why they develop will hold the key to designing good quality randomized controlled trials in the future.

After DC, cranioplasty has to be done (7) using autologous skull, or costly synthetic materials (12). Apart from its own set of complications, cranioplasty creates serious economical burden (13) in low-to-middle income countries (LMICs). They are described in detail in another chapter.

In this chapter, we classify and describe the complications of DC and suggest management techniques that can reduce the risks.

## Complications

### Decompressive craniectomy

Decompressive craniectomy has many known complications. The overall complication rates range up to 53.9% (14).

### Classification

We suggest that complications be classified as those that occur in the first 4 weeks (early) and those that manifest later (late or delayed). Early complications, which occur in the first 4 weeks, are likely to happen while the patients is still at the hospital. Specific complications tend to occur during particular time periods and awareness of that information helps anticipate and treat them efficiently. Kurland et al. classified them as (i) hemorrhagic, (ii) infectious/inflammatory, and (iii) disturbances of the CSF compartment (15). They tabulated the overall average frequency of each of the complications from a total of 142 eligible reports of thousands of patients who underwent decompressive procedures. They found that one in ten patients who underwent DC develop a complication that required additional medical and/or neurosurgical intervention.

### Timeline of various complications

Ban et al. reported, from their analysis of 89 patients, that specific complications occurred in a sequential fashion (14). Complications like cerebral contusion expansion (2.2 ± 1.2 days), newly appearing subdural or epidural hematoma contralateral to the craniectomy defect (1.5 ± 0.9 days), epilepsy (2.7 ± 1.5 days), CSF leakage through the scalp incision (7.0 ± 4.2 days), and external cerebral herniation (5.5 ± 3.3 days) occurred early. Subdural effusion (10.8 ± 5.2 days) and postoperative infection (9.8 ± 3.1 days) developed between 1 and 4 weeks postoperatively. Syndrome of the trephined and post-traumatic hydrocephalus developed after 1 month postoperatively (at 79.5 ± 23.6 and 49.2 ± 14.1 days, respectively).

### Risk factors for developing complications

Patient-specific risk factors for developing complications include poor neurological status and age. A low preoperative GCS (below eight) has been shown to increase the possibility of all types of complications (16). Age over 65 years is another risk factor (14). Though these risk factors are not modifiable, the surgical team should identify these risk groups to diligently look for emerging complications.

An overview of the complications is provided in Table 1, 2 summarizes probable causes, consequences, and management options.

**Table 1:** Overview of complications associated with decompressive craniectomy.

	**Decompressive craniectomy**
Early	•Hemorrhage (hematoma expansion) •External cerebral herniation •Wound complications •CSF leak/fistulae •Postoperative infection •Seizures/epilepsy
Late or delayed	•Subdural hygroma •Hydrocephalus •Syndrome of the Trephined

**Table 2:** Types, causes, consequences, and measures to avoid or treat complications.

**Types of complications**	**Causes**	**Consequences**	**Measures to avoid or mitigate the complication**
Expansion of conservatively managed contusions and appearance of new bleed	Loss of tamponade effect compounding the natural tendency of contusions to progress	Deterioration in sensorium, the need for evacuation	Early and more frequent scans after decompressive craniectomies at 24 and 48 h, especially in patients with contusions and contralateral calvarial fractures
Extracranial cerebral herniation	Brain edema, inadequate size of the craniectomy	Venous compromise at the edge of the craniectomy leading to further bulge and damage	Adequate size of decompressive craniectomy, re-exploration to increase the size of the decompression (rescue decompression), inserting vascular cushion at draining veins
Postoperative epilepsy	Reduced threshold for seizures but not known if the incidence is higher than if the patient has not undergone decompression. Possible effect of stretching of the scar due to sinking scalp flap	Increased metabolic demand, desaturation	Adequate dose of antiepileptic agents, early cranioplasty, as soon as possible (ASAP)
CSF leakage	Brain bulge and inability to perform watertight dural closure	Meningitis	Early detection and resuturing, water tight duraplasty
Subdural effusion	CSF flow abnormality	Usually resolves on its own	The superior and medial margin of the craniotomy should not be closer than 2.5 cm from the midline, early postoperative pressure dressing
Post-traumatic hydrocephalus	CSF flow abnormality	Deterioration, need for CSF diversion	Superior and medial margin of the craniotomy should not be closer than 2.5 cm from the midline; CSF diversion required
Postoperative neurological deterioration due to decompression	Distortion of the white matter tracts	Failure to achieve benefits of decompression	Excessively large decompression
Syndrome of the trephined	Sinking scalp flap due to lack of support and sub-atmospheric pressure causes changes in blood flow and fluid shifts	Multiple new symptoms, delayed deterioration, and failure to hold the gains of initial improvement	Early cranioplasty (ASAP), pull up with external fixator if cranioplasty cannot be done
Postoperative infection	Greater propensity for wound breakdown and CSF leaks	Greater mortality, increase in duration of hospital stay, delay in cranioplasty	Prophylactic antibiotics
Paradoxical herniation	Subatmospheric negative intracranial pressure under the sinking flap and removal of CSF, typically by lumbar puncture.	Deterioration in sensorium and new neurological deficits	Intravenous hydration, Trendelenburg position, blood patch, and early (ASAP) cranioplasty
A higher chance for injury with trivial trauma	Unprotected cranial contents when cranioplasty is delayed	Severe injuries or death	Hinge cranioplasty, early cranioplasty

## Early complications

### Hemorrhage

**Figure 1:**
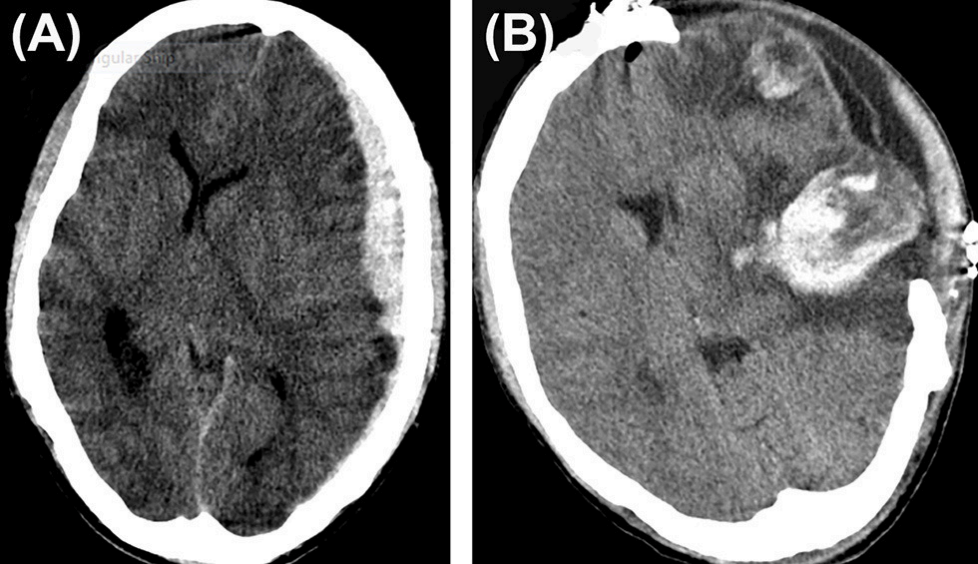
Hematoma expansion. **(A)** A case of traumatic brain injury depicting subdural hematoma **(B)**, hematoma expansion, and subdural collection post craniectomy.

Expansion of conservatively managed contusions and other bleeds are major issues that occur early after the DC (Figure 1). Most expansions occur acutely after surgery and cause clinical deterioration, prolonged hospital stay, and can even prove fatal. One theory is that the hemostatic (or tamponade) effect is lost when removing the bone, and that, along with reduction in ICP facilitates the expansion mostly on the ipsilateral side. (17–19). This hypothesis is supported by the report from Flint et al. where the propensity was higher on the side of the decompression. In their series of 40 patients, new or expanded hemorrhagic contusions were observed in 23 (58%) of 40 patients and 80% of that occurred ipsilaterally (20). Other kinds of hematomas like extradural hematomas and acute subdural hematomas can either appear *de novo* or increase in size. Expansion or evolution of new, remotely located extradural hematomas, typically occur at a fracture site (21). Expansion of hematoma contralateral or remote from the side of the craniectomy has not been commonly reported in stroke patients.

A contralateral hematoma developed an average of 2.1 days after the primary decompression surgery (16) and an ipsilateral one happened after a mean of 1.5 days (14). In the multivariate analysis of the complications in 89 consecutive patients who underwent DC, only contusion expansion led to poor outcome (14). Hemorrhagic progression of infarcts occur at a frequency of about 23.7% (123/519) of malignant stroke patients who underwent DC (15).

We suggest mandatory CT scan(s) in the first 48 h after DC to help detect this complication quickly and limit the damage.

### External cerebral herniation

**Figure 2:**
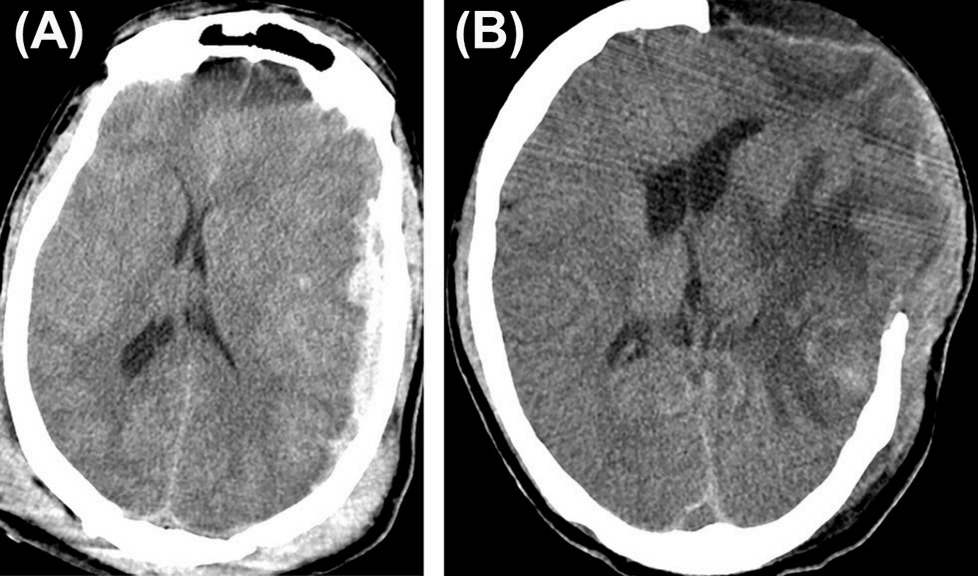
Cerebral herniation. **(A)** A case of traumatic brain injury depicting cerebral herniation **(B)** from the craniectomy site.

External cerebral herniation appears during the first week after surgery (Figure 2). Yang defined it as more than 1.5 cm of herniated brain tissue through the center of the craniectomy defect (16). The incidence is up to 25%. It is thought to be caused by the edema induced by cerebral re-perfusion and increased hydrostatic gradient from the capillaries, after decompression (17). Brain edema causes bulging of the brain and kinking of the draining veins at the edges of the craniectomy which in turn causes venous congestion, infarcts, further herniation, and brain parenchymal lacerations (22). Adequately large craniotomies and augmentative duraplasty avoid herniation (14). The Brain Trauma Foundation recommends that a large frontotemporoparietal DC (not less than 12 × 15 or 15 cm diameter) is needed over a small frontotemporoparietal DC for reduced mortality and improved neurologic outcomes in patients with severe TBI (23). Placing two small gelfoam pledgets on either side of drains at the craniectomy may prevent venous occlusion.

Paradoxical herniation is an unusual complication that tends to occur when there is negative, sub-atmospheric intracranial pressure under the caved-in scalp flap causing the brain to herniate when procedures like lumbar puncture CSF removal (24), ventriculoperitoneal shunt, subdural fluid drainage (25), or even making the patient assume a vertical position for postoperative mobilization is done (26). Intravenous hydration and Trendelenburg position has been used to successfully reverse the herniation.

### Wound complications

Wound complications following DC or cranioplasty after DC have been classified as dehiscence, ulceration, or necrosis (27). The large size of the scalp flap and the increased probability of injury to the superficial temporal artery during emergency surgery predispose the wound edges to ischemia at the posterior parietal and temporal areas. The pressure of the brain bulge aggravates the ischemia. The underlying, exposed, injured, or ischemic brain is especially vulnerable to infective complications once the wound breaks down.

Meticulously preserving the superficial temporal artery and limiting the posterior extent of the flap to no more than 5 cm behind the ear could reduce chance of ischemic flap breakdown. A retrospective comparison of patients operated using an n-shaped incision with those who were operated using the conventional question mark flap showed that the former technique could accomplish greater bony decompression, allows more brain protrusion and is faster to perform (28). We have noticed that making a retroauricular incision could also reduce flap necrosis.

### CSF leak/fistulae

The overall prevalence of CSF leak/fistulae due to DC has been shown to be up to 6.3% (15). In patients undergoing DC for cerebral venous sinus thrombosis (CVST), it was seen in 2.9% (29). It appears intuitive that a meticulous augmentative duraplasty and watertight scalp closure would prevent the exodus of CSF from the wound and reduce infection risk. However, a recent randomized controlled trial where watertight duraplasty was compared with rapid-closure DC without watertight duraplasty, there was no statistically significant difference in complications like CSF leak between the two groups of 29 patients each (30). Rapid closure DC without water tight duraplasty was on an average 31 min faster and hence cheaper. Though the authors claim that both procedures are equivalent, the trial was never powered or designed to prove non-inferiority of the test procedure and hence the results should be taken with caution (31).

### Postoperative infections

**Figure 3:**
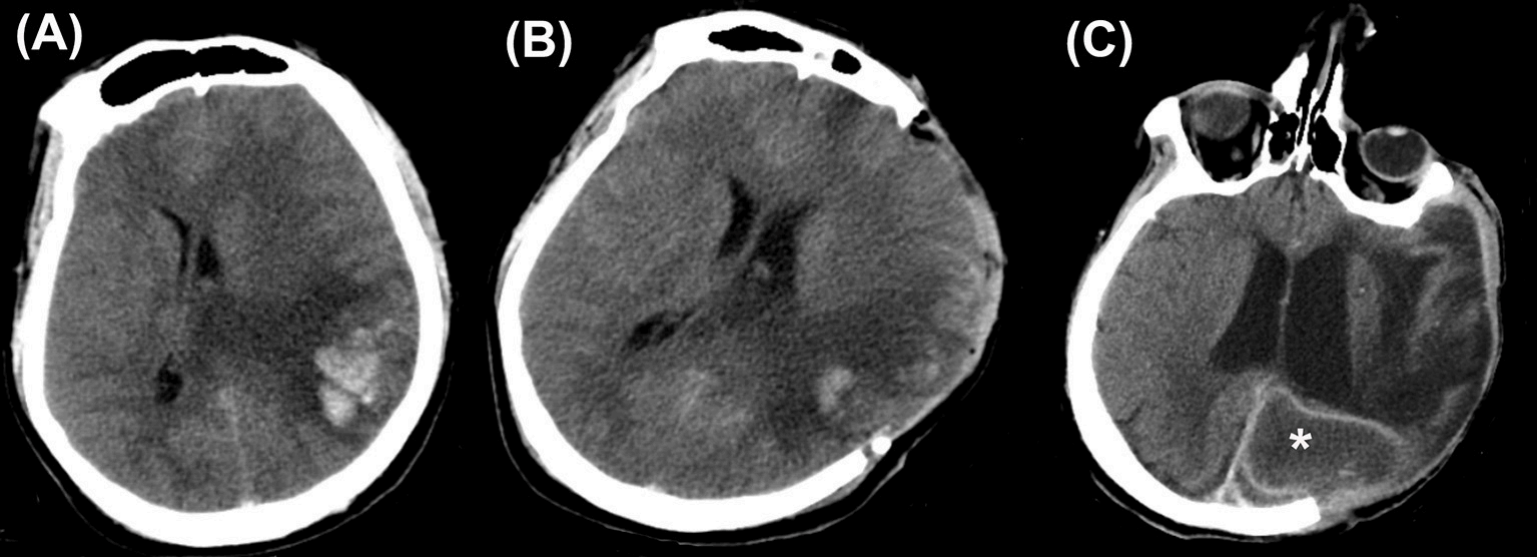
Infections. Computed tomography depicting **(A)** a case of cerebral venous sinus thrombosis. **(B)** Post craniectomy showed a reduction in the midline shift. **(C)** However, this patient developed brain abscess (asterisk) 2 months later.

Superficial wound infections including wound breakdown, necrosis, surgical site infection, sub-galeal collections, and wound breakdown occurred in about 10% of patients and incidence of deeper infections like an epidural abscess, and subdural empyema was just under 4% (15). Figure 3 shows a brain abscess which developed 2 months after DC for CVST (Figure 3C).

The incidence of meningitis and ventriculitis is 4% probably due to the higher chances of CSF leaks. Early detection by looking for signs of meningeal irritation and guarded lumbar puncture CSF analysis is warranted.

**Figure 4:**
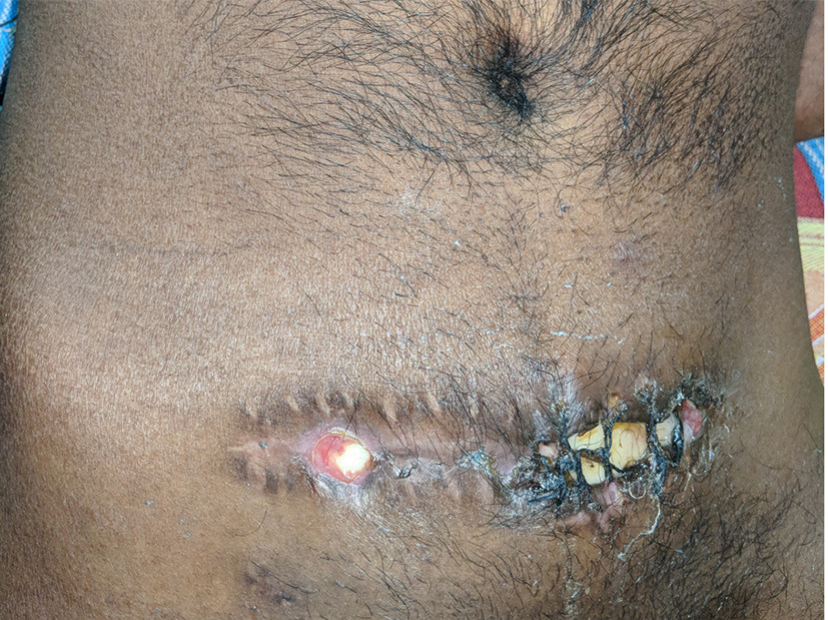
Abdominal wound infection. A partially exposed bone flap is seen through the gaped abdominal storage site, predisposing to infections.

Apart from the scalp wound complications, wound breakdown, and infection can occur when the bone flap is preserved in an abdominal pouch (Figure 4).

### Seizures/Epilepsy

Postoperative epilepsy has been documented in a varying proportion of patients who have undergone DC (14, 32–36). Suggested mechanisms include graded increases in hyperexcitability and a reduced epileptogenic threshold (14, 37). Creutzfeldt et al. retrospectively assessed 55 patients who underwent DC for malignant middle cerebral artery infarction. Of these, 49% of the patients developed seizures within the first week and 45% developed epilepsy within 1 year of surgery (32). Similarly, Santamarina et al. observed occurrence of seizures in 47.5% of all patients and in 53.7% of survivors undergoing DC for malignant MCA infarction. Logistic regression revealed that only prolonged delay from the onset of stroke to decompression (>42 h) independently predicted the development of epilepsy (34). In another study, Brondani et al. reported the prevalence of seizures in 61% (21 out of 36) of the patients with malignant MCA infarction undergoing DC. Furthermore, 59% (19 out of 34) patients developed epilepsy (33). Although a non-significant difference existed between TBI patients with or without seizures (incidence of 10.8%), the hospital stay prolonged significantly in the former group (35). Identifying the key risk factors predisposing to seizures and their effect on clinical outcomes needs more prospective studies.

In the case of TBI, Ban et al. reported that only about 3% developed seizures despite the use of anticonvulsants. Seizures disappeared in all the patients after increasing the dosage or after adding other antiepileptic drugs and that is a reasonable approach to follow in the first 2 weeks post injury (14). An early cranioplasty might serve to mitigate their occurrence, however, studies addressing this issue are currently lacking. Phenytoin and levetiracetam can be considered as antiepileptic drugs.

### Late complications

#### Subdural hygroma

Subdural hygroma formation is another widely encountered complication after DC occurring in 27.4% (723/2,643) of patients with TBI and 12.5% (42/336) of patients with malignant infarction treated with DC in the total frequency calculation done by Kurland et al. (15). The putative mechanisms seems to be due to CSF flow abnormalities that develop after decompression possibly because of a disruption of the subarachnoid CSF pathways either due to trauma or surgical manipulation (38), or due to increased cerebral perfusion pressure (39). The common locations are the subdural, subgaleal, or interhemispheric areas (16, 40, 41), Though there is a speculative relationship with the development of hydrocephalus, subdural hygromas usually resolve spontaneously. But it has been shown to be associated with a worse neurological outcome (42). Effusions are thought to be reduced by a duraplasty.

Early pressure dressing applied 7–10 days after DC has been shown to reduce this complication in a small randomized controlled trial (43). A tense collection of fluid can rarely cause pressure on the brain due to a ball valve effect and has been termed external brain tamponade and such hygromas require drainage (16, 44, 45).

#### Hydrocephalus

**Figure 5:**
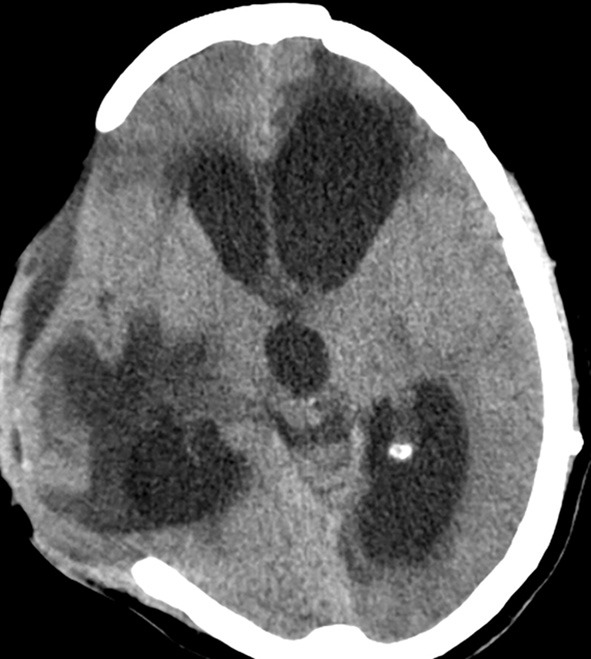
Hydrocephalus. Computed tomography depicting a case of hydrocephalus after craniectomy.

Communicating hydrocephalus is another non-trivial complication of decompressive procedures because of the perturbation of CSF flow dynamics (Figure 5). Depending on the diagnostic criteria the incidence ranges from 0.7 to 86% (42). Bonis et al. showed by logistic regression analysis that the only factor that seemed to be associated with both subdural hygroma and hydrocephalus was if the superior margin of the craniectomy was closer than 2.5 cm to the midline (42). Development of hydrocephalus is also known to predict an unfavorable outcome (46). An early cranioplasty seems to mitigate the risk of post-traumatic hydrocephalus in a retrospective cohort study of 91,583 patients <21 years with TBI, in whom 846 developed post-traumatic hydrocephalus (47). Craniectomy without early cranioplasty was associated with markedly increased adjusted odds of post-traumatic hydrocephalus (aOR 3.67, 95% CI 2.66–5.07), an effect not seen in those undergoing cranioplasty within 30 days (aOR 1.19, 95% CI 0.75–1.89).

#### Syndrome of the trephined

**Figure 6:**
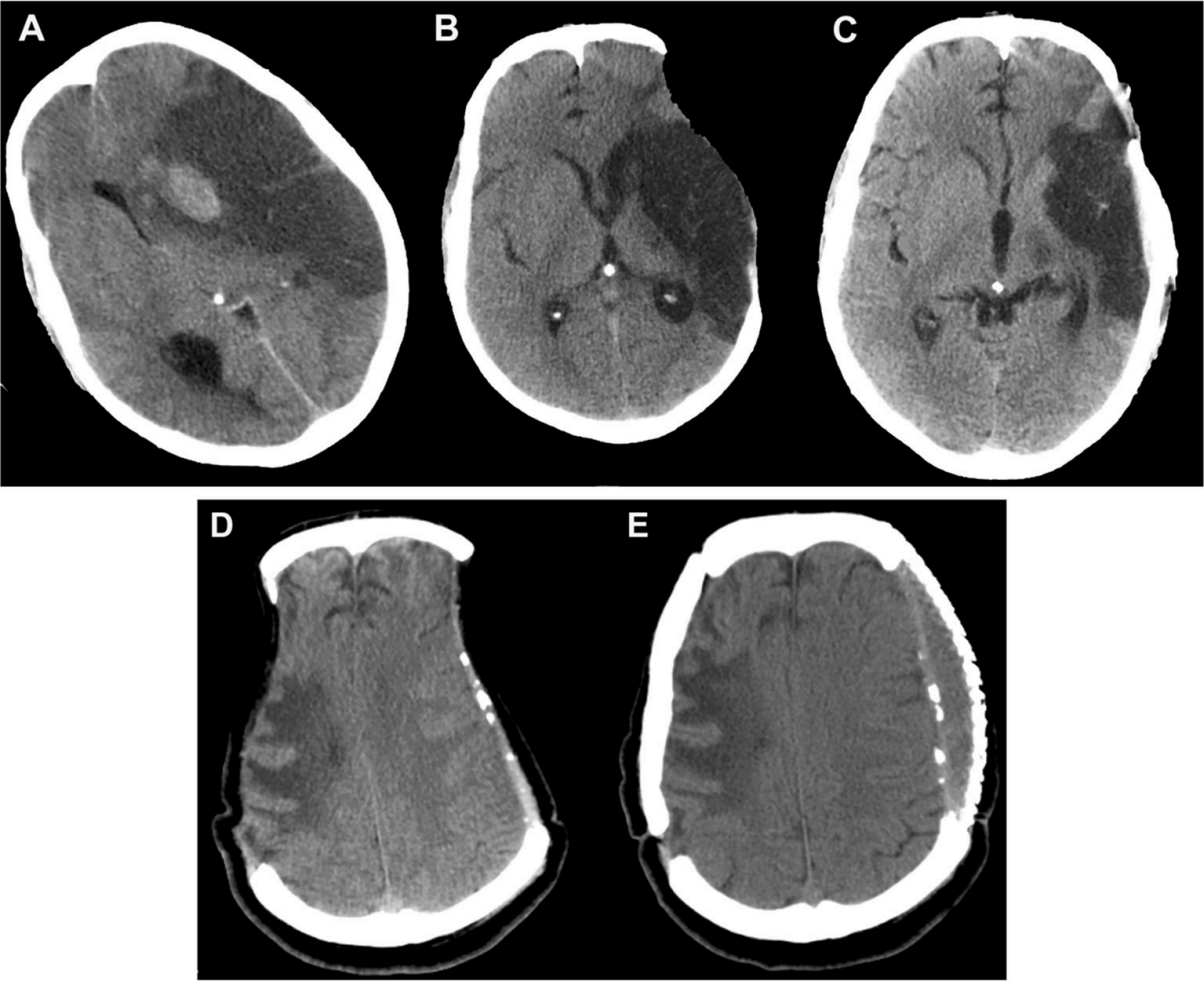
Sunken flap syndrome. Computed tomography depicting **(A)** malignant hemispheric infarction, **(B)** sunken flap syndrome after 6 months, which improved **(C)** post cranioplasty. In a different patient **(D)**, bilateral sunken flap syndrome was observed 25 months post DC, and **(E)** improved after cranioplasty. DC, decompressive craniectomy.

Syndrome of trephined has an overall prevalence of 10% (15). It was initially described by Grant and Norcross in 1939 (48). The sinking of the scalp due to lack of bony support (Figure 6) causes cerebral blood flow anomaly and dysfunction in the underlying cortex. Motor syndrome of the trephined is hypothesized to occur in patients who have had contusion induced low-density parenchymal areas. Delayed fluid shifts occur due to impaired CSF flow dynamics and this goes on to produce cerebral blood flow abnormalities and impaired motor function in a previously unaffected limb many months later (49). The syndrome can manifest in myriad ways and the most common symptoms identified in a recent systematic review were motor weakness (61.1%) followed by cognitive deficits (44.4%), language deficits (29.6%), altered level of consciousness (27.8%), headache (20.4%), psychosomatic disturbances (18.5%), seizures or electroencephalographic changes (11.1%), and cranial nerve deficits (5.6%) (50). It manifests either as new symptoms causing deterioration of the patient condition or as failure to retain the early gains. It could manifest as early as 3 days to as late as 7 years (with an average of 5 months). We must be mindful of the fact that only motor symptoms are obvious and it is quite easy to miss the diagnosis of syndrome of the trephined when non-motor symptoms like cognitive alterations occur. These symptoms, as well as, cerebral blood flow abnormalities improve dramatically after a cranioplasty. Yang has suggested it is safe to do early cranioplasty within 5–8 weeks to mitigate this risk (51) and a recent meta-analysis of observational studies involving 528 patients seems to support the possibility that neurological improvement is better in that group (52). If cranioplasty cannot be done due to a reason like infection and the patient is suffering from the effects of the sunken scalp flap, then a novel method of long standing scalp retraction using an external frame can be tried as described by Kim et al. (53).

Undue delay in cranioplasty and resorption of the bone flap after cranioplasty causes unsightly depression of the scalp. Temporal hollowing and chewing difficulty arises due to extensive dissection of the temporalis muscle to get good decompression at the temporal base. A technique of en bloc detachment and anteroinferiorly turning of the temporal muscle using a clover leaf scalp incision has been described by Missori et al., in 21 patients undergoing DC. They reported good aesthetic results and all eligible patients reported normal chewing ability (54).

## Summary

Decompressive craniectomy for intractable intracranial hypertension due to stroke or traumatic brain injury is a proven treatment for reducing mortality and there is some evidence, albeit controversial (55), that it improves the fraction of good grade survivors. But the therapy is fraught with multiple, non-trivial complications that need to be anticipated and treated early (see Table 1 for an overview). Doing a sufficiently large cranioplasty to avoid cerebral herniation and having a low threshold diagnosing for progression of bleeds in the immediate postoperative period cannot be over emphasized. An early cranioplasty, preferably within 12 weeks, as soon as the brain is lax, is advisable to prevent long-term complications of DC.

## Author contributions

MG, NS prepared, edited, structured, revised, and critically reviewed the manuscript. DS, SK, and DB critically reviewed and accepted the final draft. BD edited, critically reviewed, and accepted the final draft.

### Conflict of interest statement

The authors declare that the research was conducted in the absence of any commercial or financial relationships that could be construed as a potential conflict of interest.
